# Improving Photovoltaic Properties of P3HT:IC_60_BA through the Incorporation of Small Molecules

**DOI:** 10.3390/polym10020121

**Published:** 2018-01-26

**Authors:** Binrui Xu, Gopalan Sai-Anand, Anantha-Iyengar Gopalan, Qiquan Qiao, Shin-Won Kang

**Affiliations:** 1 School of Electronics Engineering, College of IT Engineering, Kyungpook National University, 80 Daehakro, Bukgu, Daegu 41566, Korea; kezherui123@gmail.com; 2 Global Innovative Center for Advanced Nanomaterials, Faculty of Engineering and Built Environment, University of Newcastle, Callaghan, NSW 2298, Australia; SaiAnand.Gopalan@newcastle.edu.au; 3 Future Industries Institute, Division of Information Technology, Engineering and Environment, University of South Australia, Mawson Lakes, SA 5095, Australia; 4 Research Institute of Advanced Energy Technology, Kyungpook National University, Daegu 41566, Korea; algopal99@gmail.com; 5 Center for Advanced Photovoltaics, Department of Electrical Engineering and Computer Science, South Dakota State University, Brookings, SD 570007, USA; qiquan.qiao@sdstate.edu

**Keywords:** bulk-heterojunction, polymer solar cells, morphology, additive, ternary blend

## Abstract

We investigated the role of a functional solid additive, 2,3-dihydroxypyridine (DHP), in influencing the optoelectronic, morphological, structural and photovoltaic properties of bulk-heterojunction-based polymer solar cells (BHJ PSCs) fabricated using poly(3-hexylthiophene): indene-C_60_ bisadduct (P3HT:IC_60_BA) photoactive medium. A dramatic increase in the power conversion efficiency (~20%) was witnessed for the BHJ PSCs treated with DHP compared to the pristine devices. A plausible explanation describing the alignment of pyridine moieties of DHP with the indene side groups of IC_60_BA is presented with a view to improving the performance of the BHJ PSCs via improved crystalline order and hydrophobicity changes.

## 1. Introduction

**Scheme 1:**
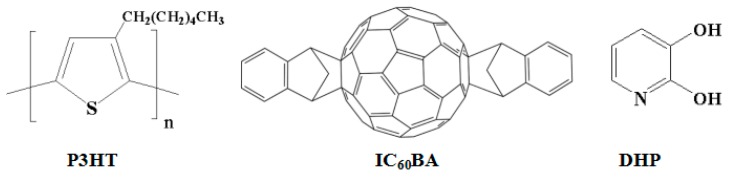
Chemical structures of P3HT, IC_60_BA and DHP.

Bulk-heterojunction polymer solar cells (BHJ PSCs) are considered prospective candidates for renewable energy technology, owing to their functionalities in new devices, and multiple advantages such as ease of fabrication, low cost, light weight, and roll-to-roll compatibility [1,2,3]. Single-junction BHJ PSCs have achieved a power conversion efficiency (PCE) in excess of 11% [4]. In the process of enhancing the PCE and overcoming the problem (a lower open-circuit voltage (*V_oc_*) of ~0.6 V) associated with the use of poly(3-hexylthiophene): [6,6]-phenyl-C_61_-butyric acid methyl ester (P3HT:PCBM)blend, Li et al. demonstrated the use of an indene-C_60_ bisadduct (IC_60_BA) as the electron acceptor in the BHJ PSCs [5]. The PSCs based on P3HT:IC_60_BA (Scheme 1) pairs showed a significant *V_oc_* of 0.84 V and a higher PCE [6,7,8]. However, further research exploring the potential of IC_60_BA to achieve high-performance PSCs is continuing [9,10,11]. The manipulation of active layer nanoscale morphology with optimal phase separation and bi-continuous interpenetrated donor/acceptor 3D networks can be achieved through the inclusion of processing additives (e.g., *n*-octylthiol [12], 1,8-diiodooctane [13], 1-chloronaphthalene [14], *N*-methyl-2-pyrrolidone [15], 1,8-octanedithiol [16], maleic anhydride [17], etc.) to accentuate the efficiency of BHJ PSCs [18]. The functional roles of the selected additives, such as their boiling point, and the nature of their interaction with the donor or acceptor in the photoactive blend, play key roles in the nanomorphology, crystallinity of the components, mobility, photocurrent, and PCE of the PSC [19,20]. A literature search reveals that the exploration of functional additives to optimize the morphology of the bis-adduct fullerene-based binary systems and optoelectronic properties has only sporadically been reported, and hence is worthy of in-depth investigation [21,22].

**Figure 1:**
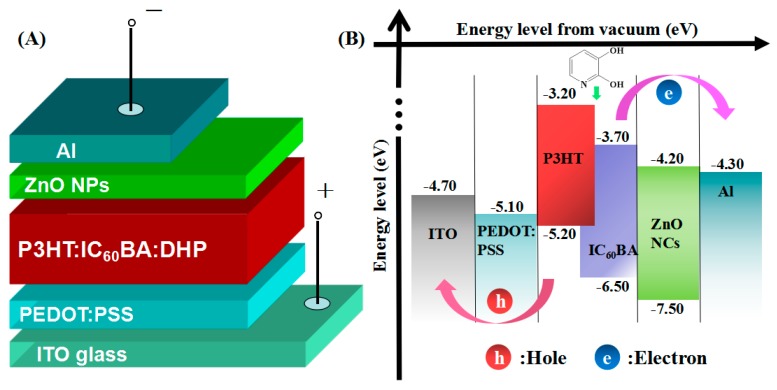
(**A**) Device schematic and (**B**) energy level band diagram.

In this work, we focus on the optimization of the P3HT:IC_60_BA blend system by introducing a functional additive, 2,3-dihydroxypyridine (DHP, shown in Scheme 1) to generate a ternary P3HT:IC_60_BA:DHP blend system [23,24,25] and improve the PV performance of BHJ PSCs in a standard device geometry: glass/ITO/poly(3,4-ethylenedioxythiophene):poly(styrene sulfonate) (PEDOT:PSS)/P3HT:IC_60_BA:DHP/zinc oxide nanocrystals (ZnO NCs)/Al (Figure 1A) [26,27,28]. The selection of DHP takes into account its chemical structure (cyclic 6-π electron and presence of an electronegative nitrogen atom in the ring) and multifunctional properties (p*K*a = 5, pH  =  8.22, a proton acceptor and intermolecular hydrogen bonding, as well as intermolecular hydrogen bond formation due to the presence of two hydroxyl groups) [29]. Therefore, it is reasonable to assume that the pyridine in DHP can form π–π interaction with the thiophene ring in P3HT, and the DHP molecule can form intermolecular hydrogen bond with IC_60_BA. These interactions could lead to optimal phase separation between P3HT and IC_60_BA, and modify the morphology of P3HT:IC_60_BA:DHP. Figure 1B depicts the energy level band gap of the studied materials. After the incorporation of DHP into P3HT:IC_60_BA system, the PCE of the fabricated BHJ PSCs with the ternary blend system exhibited a significant increase in PCE (~20%) as compared to the parent device. Herein, we present the details of our investigation of role of DHP towards influencing the optical, microstructural, morphological and PV properties of BHJ PSCs [30,31].

## 2. Experimental Section

### 2.1. Materials

PEDOT:PSS (Clevios P VP AI. 4083, H.C. Starck, Inc., Newton, MA, USA), P3HT (molecular weight (MW) = 4500, Luminescence Technology Corp., Taiwan, China), IC_60_BA (MW = 953.40, Luminescence Technology Corp., Taiwan, China), 1,2-dichlorobenzene (DCB) (99%, Sigma-Aldrich, Seoul, Korea), 2,3-dihydroxypyridine (DHP, 99%, Tokyo Chemical Industry Corp., Ltd., Tokyo, Japan).

### 2.2. Fabrication of the BHJ PSCs

P3HT, IC_60_BA and DHP were dissolved in DCB in a 1:1:0.5 weight ratio and stirred overnight at 60 °C to prepare a photoactive ink solution. The as-prepared ink was spin-coated on a pre-formed ITO/PEDOT: PSS (30 ± 5 nm) film at 600 rpm for 60 s, to fabricate a desired thickness (~250 nm) [32]. The as-cast films were then solvent-annealed in a sealed glass petri dish kept in a customized box for a few hours, and a protective layer of ZnO NCs was deposited on top to passivate the photoactive layer [8,33,34]. After edge beading with acetone, an aluminum cathode (~150 nm) was thermally evaporated onto the device stack inside a vacuum chamber. The active area of the device was 9 mm^2^. Finally, the fabricated devices were post-annealed at 150 °C for 10 min to improve the interfacial characteristics [35]. All the film deposition steps were performed in an open-air atmosphere with a relative humidity of 15–25%.

### 2.3. Measurements and Characterization

A light source integrated with a 300-W Xenon arc lamp (XES-300S1, SAN-EI Electric Corp., Osaka, Japan) with an illumination and a computer-controlled source meter (2400 series, Keithley, Inc., Seoul, Korea) were used to measure the PV performance parameters of BHJ PSCs under standard AM 1.5 G illumination (100 mW·cm^−2^). UV–Vis absorption spectra were obtained using a UV–Vis spectrophotometer (Shimadzu Corp., Kyoto, Japan). The crystallinity characteristics were studied using a X-ray diffractometer (D/Max-2500, Rigaku Corp., Beijing, China). Atomic force microscopy (AFM) images were probed to understand the modified donor-acceptor morphology using a scanning probe microscope (Model 5500, Agilent Technologies, Inc., Santa Clara, CA, USA) and analyzed with the software Park Systems XEI (Park Systems, Suwon, Korea). Photoluminescence (PL) spectra of the films were recorded using a PL spectrophotometer (Spectra pro 2150i, Acton Research Corp., Acton, MA, USA). A drop shape analyzer (Kruss, Hamburg, Germany) was utilized to measure the contact angles. The thickness of the active layer was measured by Field Emission Scanning Electron Microscopy (Hitachi SU8220, Tokyo, Japan).

## 3. Results and Discussion

### 3.1. Optical, Structural and Morphological Properties

**Figure 2:**
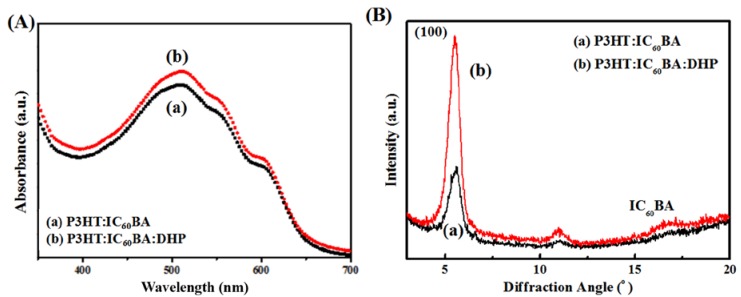
(**A**) Absorption spectra of (a) P3HT:IC_60_BA and (b) P3HT:IC_60_BA:DHP thin films, and (**B**) XRD patterns of (a) P3HT:IC_60_BA and (b) P3HT:IC_60_BA:DHP films.

The absorption spectra of P3HT:IC_60_BA and P3HT:IC_60_BA:DHP thin films are shown in Figure 2A. The characteristic vibronic peaks are observed at 510, 550 and 603 nm, while the absorbance of the P3HT:IC_60_BA:DHP was significantly enhanced in the same visible region [36,37]. The peak at 510 nm is assigned to the intrachain π–π transitions of P3HT blocks. The two vibronic shoulders at 550 nm and 603 nm can be attributed to the π–π stacking structure of P3HT [38,39]. In general, the optical absorption intensity of a conjugated polymer increases with increasing crystallinity and conjugation chain length [40]. Therefore, the observed increase in absorbance can be attributed to the higher degree of P3HT crystallinity and extended P3HT conjugation length, which is consistent with the X-ray diffraction (XRD) results [41,42]. As shown in Figure 2B, the XRD pattern exhibits a significantly enhanced diffraction peak at 2θ = 5.52°, which corresponds to the (100) reflection peak of P3HT [37]. The inclusion of DHP into P3HT:IC_60_BA resulted in a clear increase in crystallinity, structural ordering, larger P3HT crystal domain size, and possible re-distribution of the photoactive components (Figure 2B). We believe that the indene side groups in IC_60_BA align parallel to the pyridine moieties of DHP, allowing the conjugate backbone of P3HT to interact with conjugated fullerene cage. As a consequence, a more ordered and dense packing structure for P3HT can be expected.

**Figure 3:**
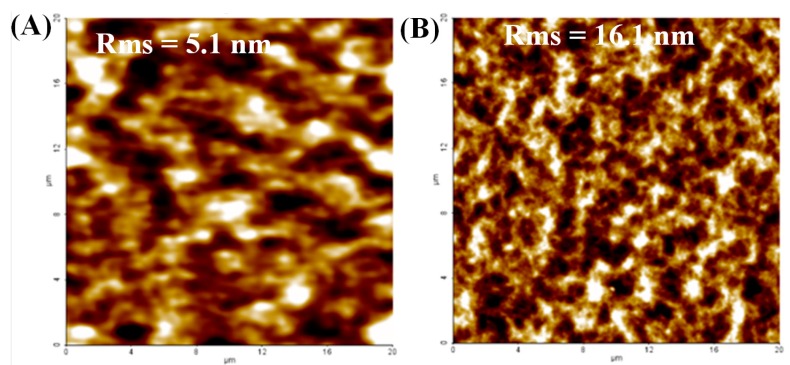

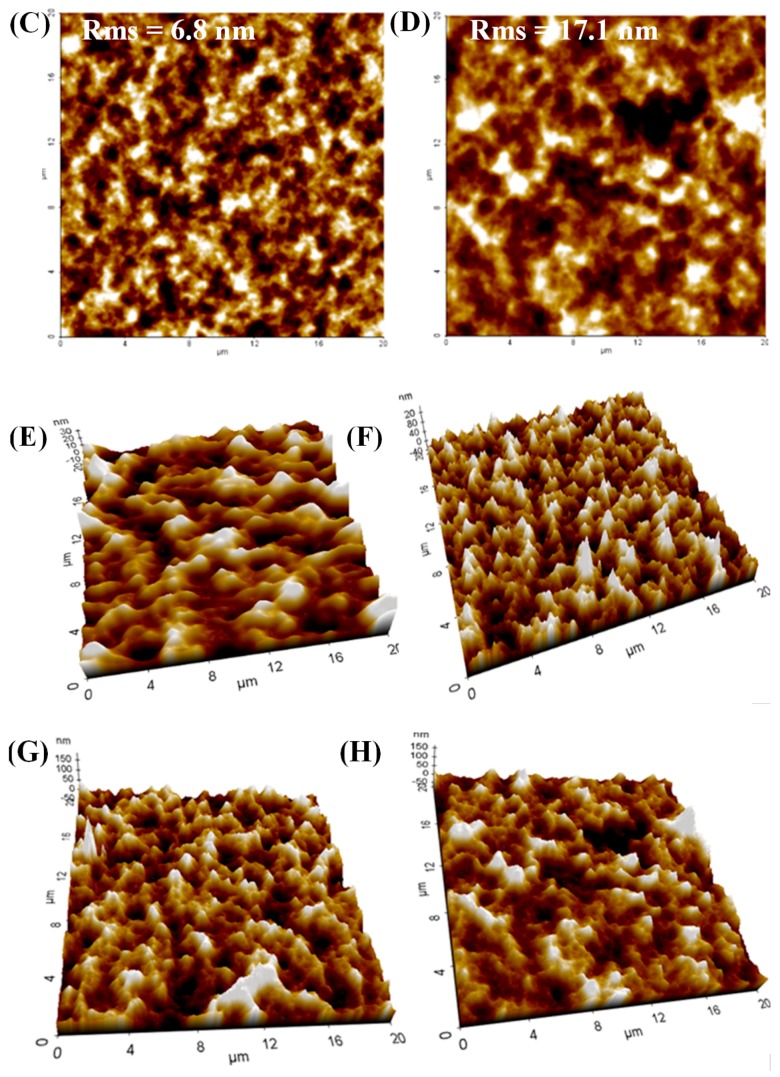
(**A**,**B**) AFM images of the P3HT:IC_60_BA and P3HT:IC_60_BA:DHP films without thermal annealing, (**C**,**D**) AFM images of the P3HT:IC_60_BA and P3HT:IC_60_BA:DPH films annealed at 150 °C for 10 min, (**E**,**F**) 3D AFM images of the P3HT:IC_60_BA and P3HT:IC_60_BA:DHP films without thermal annealing, and (**G**,**H**) 3D AFM images of P3HT:IC_60_BA and P3HT:IC_60_BA:DPH films annealed at 150 °C for 10 min.

The PV performance is related to the nanomorphology of the active layer. To investigate how the inclusion of DHP influences the active layer morphology, AFM images of the ITO/PEDOT:PSS/P3HT:IC_60_BA and ITO/PEDOT:PSS/P3HT:IC_60_BA:DHP (Figure 3A–D) and its corresponding thermally annealed counterparts (Figure 3E–H) were probed [43]. The root mean square (RMS) roughness value determined for the ternary P3HT:IC_60_BA:DHP blended film was 16.1 nm, which is higher than that of the P3HT:IC_60_BA (5.1 nm) [44]. In fact, 16.1 nm is quite close to the optimal domain size (10~20 nm) of P3HT for the improved exciton dissociation [45]. The increase in surface roughness arises from self-assembled P3HT and IC_60_BA domains due to possible molecular-level interactions with DHP and solvent annealing [41]. In the presence of the DHP, optimal phase separation was achieved with a 3D interpenetrating network, enabling improved exciton dissociation and charge transport [46,47]. Compared to the film without DHP, the RMS roughness of the modified film increased from 6.8 nm to 17.1 nm after annealing at 150 °C for 10 min. While the surface of the P3HT:IC_60_BA film is richer in P3HT, the top surface of P3HT:IC_60_BA:DHP film is dominated by the presence of IC_60_BA domains. The preferential existence of IC_60_BA at the surface of P3HT:IC_60_BA:DHP would enhance the hydrophobicity at the interface. 

**Figure 4:**
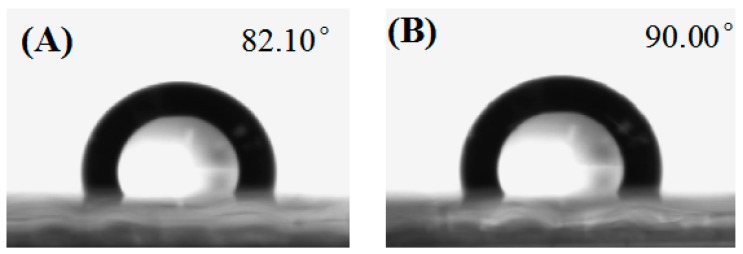
Water droplet contact angles of (**A**) P3HT:IC_60_BA and (**B**) P3HT:IC_60_BA:DHP.

We investigated the surface characteristics through water contact angle measurements (Figure 4). The P3HT:IC_60_BA:DHP surface is relatively hydrophobic, giving rise to a contact angle of 90.0°, while the surface of P3HT:IC_60_BA is less hydrophobic with a contact angle of 82.1°. The literature suggests that the surface tension of P3HT is much lower than that of IC_60_BA. These two factors are in accordance with the predominance of fullerene (IC_60_BA) rich phase at the P3HT:IC_60_BA:DHP surface [48]. The increased contact angle of the P3HT:IC_60_BA:DHP confirms the improved hydrophobicity and re-distributions of the polymer and fullerene components in the ternary blended film. We attribute that the entropic effects resulted from the differences in the flexibilities of P3HT chains and crystallization behavior lead to the preferential segregation of IC_60_BA at the top surface of DHP treated active layer.

### 3.2. Photovoltaic Properties

**Figure 5:**
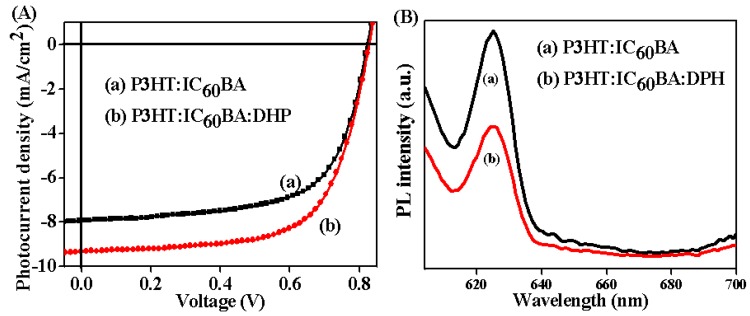
(**A**) J–V characteristics of the BHJ PSCs fabricated with (a) P3HT:IC_60_BA and (b) P3HT:IC_60_BA:DHP-based BHJ PSC, and (**B**) PL spectra of (a) P3HT:IC_60_BA and (b) P3HT:IC_60_BA:DHP thin films.

To validate the efficiency of the ternary P3HT:IC_60_BA:DHP blend system, current density-voltage (J–V) curves were recorded under AM 1.5 G illumination at 100 mW·cm^−2^ and the performance of the BHJ PSCs with and without the additive are shown in Figure 5A, Table 1. The pristine device without DHP exhibited a PCE of 4.22%, with an open-circuit voltage (*V_oc_*) of 0.83, a short current density (*J_sc_*) of 8.36 mA·cm^−2^, a fill factor (FF) of 0.63, and a series resistance (*R_s_*) of 160 Ω. By introducing DHP into P3HT:IC_60_BA, PCE, *J_sc_* and FF were significantly improved and *Rs* relatively decreased, while *V_oc_* nearly maintained the same value compared to the pristine device. The best PCE of 5.06% was achieved at the optimized weight ratio of 0.5 wt % DHP into the P3HT:IC_60_BA, with *V_oc_*, *J_sc_*, FF and *R_s_* values of 0.83 V, 9.39 mA·cm^−2^, 0.66 and 129 Ω, respectively [49,50]. The observed improvement in the PCE is mainly related to the enhanced *J_sc_* and reduced *R_s_*, which are ascribed to the optimized morphology, enhanced crystallinity of P3HT and improved light absorption of P3HT:IC_60_BA film with the addition of 0.5 wt % DHP.

In order to investigate charge separation and recombination dynamics of the active layer, steady state PL spectra of the P3HT:IC_60_BA and P3HT:IC_60_BA:DHP films (Figure 5B) were acquired by exciting at 600 nm [51,52]. The peak due to P3HT at ~624 nm is significantly quenched for P3HT:IC_60_BA:DHP as compared to the P3HT:IC_60_BA, resulting in efficient exciton dissociation between P3HT and IC_60_BA [53]. Furthermore, the space charge limited current (SCLC) method was used to confirm the improved hole mobility of P3HT:IC_60_BA and P3HT:IC_60_BA:DHP-based devices [54,55]. With the fabricated hole-only devices (glass/ITO/PEDOT:PSS/active blend/Au), the hole mobility values of binary P3HT:IC_60_BA and ternary P3HT:IC_60_BA:DHP blends were calculated as 2.15 × 10^−4^ and 8.56 × 10^−4^ cm^2^·(Vs)^−1^, respectively [56]. Therefore, the addition of DHP in P3HT:IC_60_BA facilitates balanced carrier mobility as compared to P3HT:IC_60_BA [57].

## 4. Conclusions

In summary, the BHJ PSCs fabricated with the P3HT:IC_60_BA active layer containing 0.5 wt % DHP exhibited a remarkable (~20%) increase in the PCE as compared to PSC based on the control devices. This improvement is attributed to the increased crystallinity, as well as the synergistic influence of the improved optical, microstructural, and optoelectronic properties, of the photosensitive layer. This study provides an inspiration and scope for the researchers aiming to improve the overall efficiency of any BHJ PSC system through a judicious selection of components (additives/dopants etc.) having multifunctional capabilities.

## Figures and Tables

**Table 1:** Summary of the photovoltaic performance parameters of the BHJ PSCs fabricated with P3HT:IC_60_BA and P3HT:IC_60_BA:DHP. The average PCE values were obtained from eight independent devices.

BHJ PSC	*V_oc_* (V)	*J_sc_* (mA/cm^2^)	*R_s_* (Ω)	FF	PCEE (%)
P3HT:IC_60_BA	0.83 ± 0.013	8.36 ± 0.32	160 ± 23.98	0.63 ± 0.020	4.22 ± 0.13
P3HT:IC_60_BA:DHP	0.83 ± 0.03	9.39 ± 0.09	129 ± 4.04	0.66 ± 0.006	5.06 ± 0.05

## Supplementary Materials

The following are available online at http://www.mdpi.com/2073-4360/10/2/121/s1, Figure S1: FE-SEM cross-section image of the completed device with the structure of glass/ITO/PEDOT:PSS/P3HT:ICBA:DHP/ZnO NCs/Al.
