# Supplementary material for: Improving Photovoltaic Properties of P3HT:IC60BA through the Incorporation of Small Molecules
Source: Polymers (Basel). 2018 Jan 26;10(2):121. doi: 10.3390/polym10020121 (PMC6415164; doi:10.3390/polym10020121)
Supplement: Supplementary file 1 [file polymers-10-00121-s001.pdf]

Supplementary information for

Improving Photovoltaic Properties of P3HT:IC_{60}BA Through the Incorporation of Small Molecule

Binrui Xu [1,†], Gopalan Sai-Anand [2,3,†], Anantha-Iyengar Gopalan [4], Qiquan Qiao [5] and Shin-Won Kang [1,*]

1 School of Electronics Engineering, College of IT Engineering, Kyungpook National University, 80 Daehakro, Bukgu, Daegu 41566, Korea; kezherui123@gmail.com
2 Global Innovative Center for Advanced Nanomaterials, Faculty of Engineering and Built Environment, University of Newcastle, Callaghan, NSW 2298, Australia; SaiAnand.Gopalan@newcastle.edu.au
3 Future Industries Institute, Division of Information Technology, Engineering and Environment, University of South Australia, Mawson Lakes, SA 5095, Australia
4 Research Institute of Advanced Energy Technology, Kyungpook National University, Daegu 41566, Korea; algopal99@gmail.com
5 Center for Advanced Photovoltaics, Department of Electrical Engineering and Computer Science, South Dakota State University, Brookings, SD 570007, USA; qiquan.qiao@sdstate.edu
* Correspondence: swkang@knu.ac.kr; Tel.: +82-053-950-6829
† These authors contributed equally to this work.

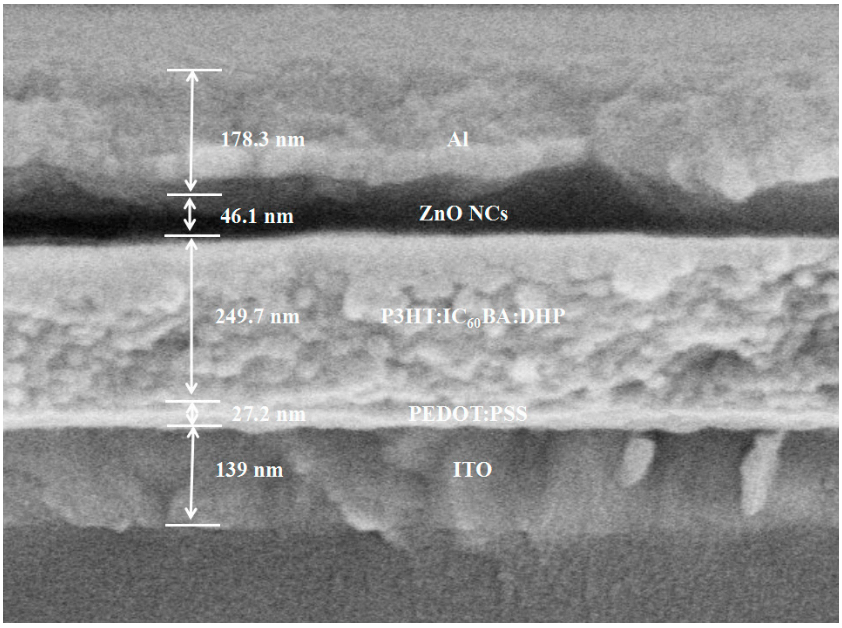

Figure S1. FE-SEM cross-section image of the completed device with the structure of glass/ITO/PEDOT:PSS/P3HT:ICBA:DHP/ZnO NCs/Al.

Figure S1 exhibited the Field Emission Scanning Electron Microscopy (FE-SEM) cross-section image of fabricated device with the structure of glass/ITO/poly(3,4-ethylenedioxythiophene):poly(styrene sulfonate) (PEDOT:PSS)/ poly(3-hexylthiophene): indene-C_{60}bisadduct: 2,3-dihydroxypyridine (P3HT:IC_{60}BA:DHP)/zinc oxide nanocrystals (ZnO NCs)/Al. It can be seen from Figure S1,

the thickness of P3HT:IC_{60}BA:DHP is near 250 nm which is belong to the desired thickness range (200 ~ 250 nm) to obtain reasonable performance of the fabricated BHJ-PSCs based on P3HT:IC_{60}BA blend system [1] [2] [3].

References

1. Tzabari, L.; Wang, J.; Lee, Y.-J.; Hsu, J.W.P.; Tessler, N. Role of charge transfer states in p3ht-fullerene solar cells. *The Journal of Physical Chemistry C* **2014**, *118*, 27681-27689.
2. Sánchez, J.G.; Ferré-Borrull, J.; Marsal, L.F.; Pallarès, J.; Balderrama, V.S.; Estrada, M. In *Performance comparison of bhj organic solar cells based on p3ht with pc70bm and icba*, 2015 IEEE International Autumn Meeting on Power, Electronics and Computing (ROPEC), 4-6 Nov. 2015, 2015; pp 1-4.
3. Nozik, A.J.; Conibeer, G.; Beard, M.C. *Advanced concepts in photovoltaics*. Royal Society of Chemistry: 2014.
